# Combined Exposure to Multiple Metals and Kidney Function in a Midlife and Elderly Population in China: A Prospective Cohort Study

**DOI:** 10.3390/toxics11030274

**Published:** 2023-03-17

**Authors:** Tianci Wang, Liming Zhang, Yujie Liu, Jian Li, Guochong Chen, Hui Zhou, Lugang Yu, Zhongxiao Wan, Chen Dong, Liqiang Qin, Jingsi Chen

**Affiliations:** 1 Department of Nutrition and Food Hygiene, School of Public Health, Suzhou Medical College of Soochow University, Suzhou 215123, China; 2 Suzhou Municipal Center for Disease Control and Prevention, Suzhou 215007, China; 3 Suzhou Industrial Park Centers for Disease Control and Prevention, Suzhou 215021, China

**Keywords:** environmental epidemiology, plasma metal, renal function, chromium, potassium, selenium

## Abstract

[Background] Metal exposure is suspected to be correlated to kidney function. However, the combined effects of co-exposing to multiple metals, especially both toxic and protective metals, have not been completely evaluated. [Method] A prospective cohort study was conducted with the “135” cohort for the evaluation of how plasma metal levels are correlated to kidney function in a midlife and elderly community in southern China. An amount of 1368 subjects without kidney disease at baseline were enrolled in the final analysis. By using linear regression and logistic regression models, the correlation of individual metal values with renal function parameters was assessed. Measuring of the multiple metal exposure level was performed by principal component analysis (PCA). [Results] Diminished renal function, as evaluated based on fast kidney function decline, or estimated glomerular filtration rate (eGFR) < 60 mL/min/1.73 m^2^, was positively associated with the plasma concentrations of chromium and potassium, but it was negatively associated with selenium and iron (*p* < 0.05). In multiple-metal analyses, linear and logistic regression models showed that the iron and chromium exposure pattern had a protective effect on renal function, whereas the sodium and potassium exposure pattern and the cadmium and lead exposure pattern increased the risk for fast kidney function decline, and eGFR < 60 mL/min/1.73 m^2^. [Conclusions] Certain metals, including chromium, potassium, selenium, and iron, were correlated with kidney function in a midlife and elderly community in China. In addition, the potential combined influences of co-exposing to multiple metals were observed.

## 1. Introduction

Chronic kidney disease (CKD) has turned into a critical and ever more prevalent global public health concern among adults; its main characteristic is impaired kidney function [1,2]. From 1990–2017, CKD incidence increased by 29.3% worldwide [3]. In China, the National Survey revealed the increase in the overall CKD incidence to be up to 10.8% in 2010, reaching almost 119.5 million CKD patients in 2012 [4]. The 2017 disease burden report referred to CKD as a leading cause of years of life lost (YLL), with 264 YLL per 100,000 population [3]. Thus, CKD is associated with health and economic issues that burden society.

In spite of CKD-related high incidence and disease burden, the risk factors for the incidence of CKD remain unclear. Underlying disease (such as diabetes, hypertension, dyslipidemia, and obesity), advanced age, lifestyle (smoking), and nephrotoxins are widely acknowledged risk factors for CKD [5]. A growing body of evidence suggests the independent relevance of exposure to certain metals in the occurrence and development of CKD [6,7,8].

Exposure to toxic metals and the excess of some essential metals might be harmful to renal function. Cadmium, a well-known toxicant, has nephrotoxicity effects that could accumulate in the kidney, leading to tubular impairment [9]. Epidemiologic evidence has shown that, even when exposed to low cadmium levels from the surrounding environment, patients had a correlation to a low estimated glomerular filtration rate (eGFR) [10]. Epidemiology studies found that exposure to lead, chromium, zinc, sodium, and potassium may also take part in the risk of renal dysfunction [11,12,13]. In contrast to that of cadmium, data on the nephrotoxicity of exposing to low levels of these metals from the environment is still limited.

Conversely, some important metals with antioxidant properties have been suggested to be capable of improving renal function. Observational results and a randomized controlled trial showed that low selenium levels had a relation to declined kidney function, and supplementation with coenzyme Q10 and selenium resulted in significant improvement in kidney function [14]. A cross-sectional study conducted among the elderly in longevity areas in China demonstrated that the adjusted OR for CKD was 0.36 (0.19, 0.65) for the greatest quartile of iron than the lowest quartile [15]. A three-wave repeated-measurement research of 201 elders revealed urinary copper was positively associated with urinary albumin-to-creatinine ratio [16].

Notably, certain metals (lead, cadmium, chromium, copper, zinc, selenium, magnesium, iron, calcium, sodium, and potassium) [17,18,19,20] may share common mechanisms, such as oxidative stress, no matter their type of effects. In vivo and in vitro studies demonstrated that toxic metals induced oxidative stress in the kidney [17], whereas antioxidant metals have been suggested to be biologically vital in defending against oxidative damage [21]. The mechanisms shared between these different metals create the risk evaluation of co-exposure to multiple metals that are critical given the chronic exposure of the public to several coexisting metals in the environment. Nevertheless, little is known regarding how exposure to multiple metals is correlated with renal function.

In this work, we used data from a prospective cohort study on the middle-aged and elderly residents of Suzhou in China in evaluating the correlations of plasma metals to diminished renal function. Furthermore, we utilized principal component analysis (PCA) to identify the health risks of co-exposure to multiple metals to kidney function.

## 2. Materials and Methods

### 2.1. Study Population

Data of the “135” cohort were utilized, a prospective cohort study conducted in the Industrial Park of Suzhou (Suzhou, Jiangsu Province, China). An amount of 7866 adults aged ≥18 years old were initially enrolled via hospitals and health examination centers in 2013, which represented about 65% of the invited residents [22,23,24]. As blood samples collected in 2013 were insufficient for the measurement of metal concentration, we excluded all data in 2013, and we used the data (demographic characteristics, clinical parameters, and plasma metal concentrations) in 2015 as a baseline (*n* = 4350). Face to face questionnaire surveys were conducted by trained investigators, and blood samples were collected for measurements of clinical and metal exposure parameters in 2015. Participants had an invitation to the follow-up examination in 2019, and 2487 aged ≥45 years old participants performed questionnaires and clinical examinations. There were 1368 residents enrolled in the final analyses. The other 1119 were excluded, including 49 lacking demographic characteristics or health status information, 363 with malignant tumors, kidney disease, and severe mental disease at baseline, as well as 707 with insufficient blood samples for detection of metals exposure (Supplementary Figure S1). Participants obtained written approval. The Helsinki Declaration was followed throughout the study with the approval of the ethics committee of Soochow University (ECSU-2010-002) for the protocol.

### 2.2. Exposure Measurement

Collecting the peripheral venous blood samples (2015) was performed utilizing ethylenediamine tetraacetic acid (EDTA) anticlotting tubes and followed by centrifugation and freezing within two hours and −80 °C storage. An inductively coupled plasma mass spectrometer (ICP-MS) and inductively coupled plasma (ICP) were utilized to assess plasma levels of 11 metals. In view of the previous studies [25] on the measurement of plasma metals, we applied a similar experimental method in this study. Briefly, the plasma samples were vortex-mixed before thawing. Then, 0.3 mL plasma samples were transferred to digestion tubes and spiked with 0.5 mL 65% HNO_3_, 1 mL 30% H_2_O_2_, and 5 mL ultrapure water. Next, they were digested for 65 min. After sample digestion, the residue was reconstituted with 10 mL ultrapure water followed by passing it through a 0.22 mm filter, and it underwent analysis by ICP-MS (NexION™ 350X, PerkinElmer Inc., Waltham, MA, USA) for lead, cadmium, chromium, copper, zinc, selenium, magnesium, iron, and calcium, as well as by ICP (Avio™ 200, PerkinElmer Inc., Waltham, MA, USA) for sodium and potassium.

During the measurement, we randomly measured case and control samples to achieve the effect of the blind method. A blank sample was analyzed in each batch (30 samples) to ensure the absence of contamination. We used internal (In) and external standard method in the determination. Spiked pooled plasma samples (collected from 200 samples randomly) were used to assess to what extent this method is precise and accurate. The validation for the accuracy of the method has been shown in Supplementary Table S1. The recovery rates of 11 metals were 89–110%, and the relative standard deviation (RSD) was 1.2–2.0%. Concentrations of metals under the limits of detection (LOD) were expressed as LOD/2.

### 2.3. Kidney Outcomes Measurement

An amount of 5 mL free-anticoagulant blood samples (collected in 2015 and 2019) underwent 10 min centrifugation and were sent to the affiliated hospital of Soochow University for creatinine determination. The Chronic Kidney Disease Epidemiology Collaboration (CKD-EPI) equation was utilized to calculate eGFR according to serum creatinine, age, and gender [26]. The CKD-EPI equation has significant precision and less bias in eGFR than the Modification of Diet in Renal Disease (MDRD) equation [27,28]. The creatinine (in mg/dL) equation was: eGFR = 141 × (Scr/κ)^α^ × 0.9929^Age^ (×1.018 if female), where κ is 0.7 if woman and 0.9 if man. In females, α is −0.329 if Scr ≤ κ and α is −1.209 if Scr > κ. In males, α is −0.411 if Scr ≤ κ, and α is −1.209 if Scr > κ. According to previous studies, the definition of fast kidney function decline was stated as a yearly decline in eGFR ≥ 5 mL/min/1.73 m^2^ [29]. Subjects with eGFR < 60 mL/min/1.73 m^2^ were classified as eGFR decline events [12].

### 2.4. Covariates

Trained professionals used semi-structured questionnaires to collect data on demographic features (e.g., age, gender, and marital status), lifestyle (e.g., smoking and drinking status), and past medical history (coronary heart disease, stroke, hypertension, diabetes, nephritis, etc.). The medical staff then conducted a physical examination of each subject (such as blood pressure, weight, height, and waist circumference). Autoanalyzer (Olympus AU640) was utilized in measuring total cholesterol (TC), triglycerides (TG), high-density lipoproteins (HDL), low-density lipoproteins (LDL), and fasting blood glucose (FPG). Calculation of body mass index (BMI) was reported as weight (kilograms) divided by the square of height (meters).

### 2.5. Statistical Analyses

SPSS (v24.0; IBM SPSS) was utilized for conducting statistical analyses with a statistically significance result regarded as two-sided *p* value < 0.05. The representation of continuous variables was reported as mean ± standard deviations (SD) or median and inter-quartile range (IQR), as shown in the distribution, while numbers and percentages (%) were given for categorical variables. By using the Mann-Whitney U test or Student’s *t*-test, *p* values of continuous variables were analyzed. By chi-square test, *p* values of categorical variables were analyzed.

A two-step process was employed for exploring the connection between metal levels and kidney function.

In the first step, two models were utilized for assessing the influences of single metals on renal function. The Mann-Whitney U test was employed for the exploration of the variation in plasma concentrations of 11 metals between eGFR groups. By using linear regression models, the association between baseline plasma metal levels and annual eGFR changes was assessed. Because of the skewed distribution, plasma metal concentrations were naturally log-transformed. Covariates within this study were in accordance with previous data that may be the potential confounders. The initial regression model did not adjust factors, while the subsequent regression model further adjusted age, gender, baseline eGFR, BMI, smoking status, and drinking status.

As metals were correlated (Supplementary Table S2), we used PCA to create summarization measures of co-exposure to multiple metals. Briefly, the Kaisere-Meyere-Olkin test was firstly utilized to study the adequacy of data of PCA; next, the PCA was employed for the naturally log-transformed levels of 11 metals to extract principal components (PCs), and the element values for every subject were calculated, showing how deeply plasma metals conform to identify exposure patterns. Then, PCs as quartiles were modeled into the linear regression model to assess its correlation to annual eGFR decline and into the logistic regression models to assess its association with fast kidney function decline and eGFR < 60 mL/min/1.73 m^2^.

## 3. Results

This study included 1368 participants, of whom 55.1% were male (Table 1). The participants had an average age of 57.6 years and an average eGFR of 91.6 mL/min/1.73 m^2^ at baseline. Throughout the duration of the follow-up, the average eGFR annual decline was 2.9 mL/min/1.73 m^2^. Individuals with fast decline in kidney function had higher concentrations of TC and LDL and lower HDL at baseline than those without. The likelihood of participants having eGFR < 60 mL/min/1.73 m^2^ was being more aged; females had higher SCR, TC, and LDL and had lower eGFR at baseline than those without having high eGFR values. Meanwhile, individuals with eGFR < 60 mL/min/1.73 m^2^ experienced a steeper annual decrease in eGFR (*p* < 0.05). Supplementary Table S3 showed that the demographic features of the included and excluded participants were compared. The included subjects tended to be male and had higher BMI than the excluded subjects.

The differences in plasma metal concentrations between eGFR groups are presented in Table 2. We found that participants with fast kidney function decline had higher plasma concentrations of chromium, sodium, and potassium and lower copper, selenium, iron, and calcium levels than the other participants. Meanwhile, after Bonferroni correction, we revealed that the plasma concentrations of eGFR < 60 mL/min/1.73 m^2^ cases were high in chromium and potassium, but lower in selenium and iron, than the other participants.

**Figure 1:**
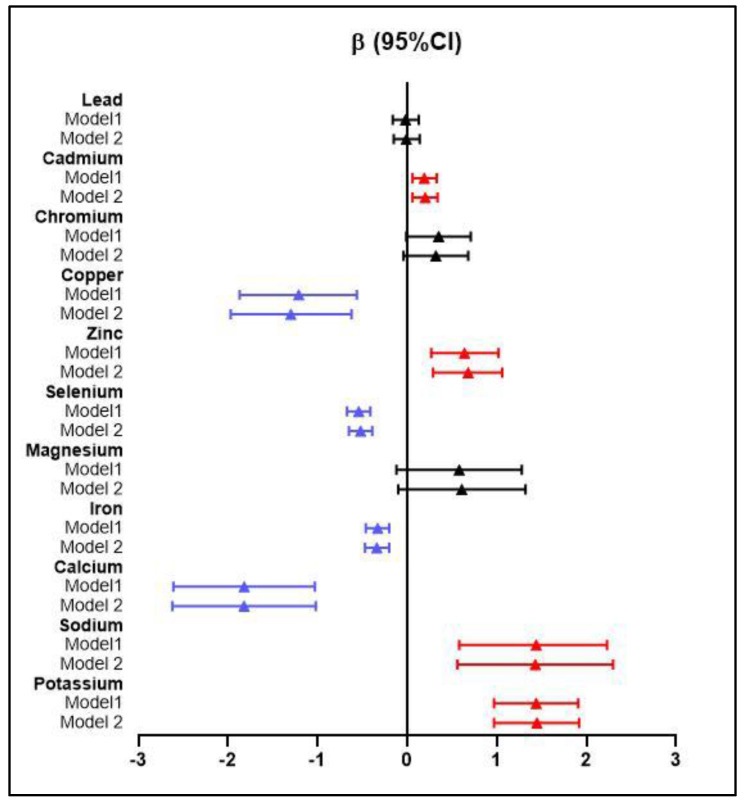
Difference (95%CI) in a yearly decline of eGFR (mL/min/1.73 m^2^) correlated to baseline plasma metal levels in the linear regression model. CI: confidence interval. Model 1:crude model; Model 2: adjusted for age, gender, baseline eGFR, BMI, smoking status, and drinking status. The inclusion of plasma metal levels in models as natural log-transformed continuous variables. *p* values were Bonferroni corrected. Red line: Bonferroni-corrected positive significance Blue line: Bonferroni-corrected negative significance.

Linear regression models (Figure 1) illustrated that the decline in annual eGFR was positively correlated with the plasma concentrations of cadmium, zinc, sodium, and potassium, as well as negatively correlated with copper, selenium, iron, and calcium.

We performed PCA to create new variables for the measurement of co-exposure to multiple metals to further explain the obtained outcomes from single-metal analyses. The four extracted PCs with eigenvalues > 1 accounted for 67.82% of the total variance. Calcium, copper, and magnesium were the major contributors to PC1, iron and chromium were the major contributors to PC2, sodium and potassium were the major contributors to PC3, and cadmium and lead were the major contributors to PC4 (Supplementary Figure S2). Among these four PCs, PC2, PC3, and PC4 had a significant correlation to the annual decline in eGFR (Table 3). In PC2, the annual increase in eGFR shown by the highest quartile group was 1.54 (1.04, 2.03) mL/min/1.73 m^2^, more than exhibited through the lowest group. The annual decrease in eGFR presented by the highest quartile group was 2.02 (1.53, 2.51) and 1.60 (1.10, 2.09) mL/min/1.73 m^2^, more than demonstrated through the lowest quartile groups in PC3 and PC4, respectively. In addition, we observed that the annual decline in eGFR tended to decrease with the increase in PC2 and tended to increase with PC3 and PC4 (*p* < 0.001).

Then, we conducted a logistic regression analysis to further explore whether PCs were related to fast decline in kidney function and eGFR < 60 mL/min/1.73 m^2^ (Table 4). After adjustments for all confounders, individuals in the greatest quartile of PC2 had less diminished renal function assessed by fast decline in kidney function or eGFR < 60 mL/min/1.73 m^2^. By contrast, participants with higher scores for PC3 and PC4 had a significant risk of a kidney function decline (*p* < 0.05).

## 4. Discussion

We found that the decline in kidney function assessed on the basis of fast kidney function decline or eGFR < 60 mL/min/1.73 m^2^ was correlated positively with plasma chromium and potassium levels and negatively correlated with selenium and iron. In addition, we identified relationships among co-exposure to multiple metals and kidney function and found that (a) the iron and chromium exposure pattern was negatively correlated with the decline in kidney function, and (b) the potassium and sodium exposure pattern and the cadmium and lead exposure pattern were positively correlated with kidney function decline.

### 4.1. Risk Factors for the Decline in Kidney Function

Potassium, the second most abundant essential metal in humans, is vital in maintaining cell function. However, the excess of some essential metals has also been suggested to be harmful to health. A large healthcare-based observational investigation in Sweden revealed that individuals with CKD had progressively higher plasma K^+^ concentrations than those without [30]. Patients with CKD easily develop hyperkalemia because the main renal organ (kidney) has a crucial role in K^+^ homoeostasis [31]. However, whether potassium accumulation could influence clinical renal outcomes is poorly known. Our findings, based on a prospective cohort, expanded previous evidence with the novel demonstration that potassium exposure may also be the cause of decline in renal function.

Chromium occurs naturally in the Earth’s outer shell and is ubiquitous in the surrounding as a result of human activities. Within the overall population, the primary inhalation of chromium by smoking or vaporization, ingestion of contaminated food and drink, and skin contact are all causes of exposure to chromium [32]. In the present study, chromium concentrations were significantly higher in cases with fast kidney function decline or eGFR incident < 60 mL/min/1.73 m^2^ than in those without. These findings were consistent with previously reported results. For example, an analysis based on the data from the National Nutrition and Health Survey in Taiwan (2005–2008) pointed out that that doubling of urinary chromium levels caused eGFR to decrease by −5.99 (−9.70, −2.27) mL/min/1.73 m^2^ [33]. A cross-sectional investigation on pediatric patients with CKD found that those with elevated plasma levels of vanadium and chromium may have low eGFR [34]. Another cohort study that recruited 934 participants with essential hypertension observed that exposure to the surrounding chromium and uranium contributed to the eGFR decline [35].

### 4.2. Protective Factors for the Decline in Renal Function

Selenium is universally present as an antioxidant in nature and in organisms. The selenium median concentration in our population study was 22.0 µg/L, which was considerably lower than that in populations in the south (102.27 µg/L) [11] or central (65.2 µg/L) [12] China population, German adults (61.38 µg/L) [36], and Latin American adults (91.51 µg/L) [37], but was similar to those in populations in areas of selenium deficiency in China (Xichang: 27 µg/L) [38]. Our present study identified a negative association between plasma selenium and the risk of diminished kidney function in accordance with previous studies conducted on areas of selenium deficiency or adequacy, but not in areas of selenium excess [39,40,41]. Only two studies found a positive relation between selenium and serum uric acid values in populations with selenium intakes above the recommended level or high selenium exposure levels (median concentration: 102.27 µg/L) [11,42]. A growing number of observational studies recently reported a U-shaped dose–response connection between selenium status and health outcomes (bone mineral density, blood pressure, and fasting plasma glucose) [43,44]. Whether U-shaped associations also exist in the relationship between selenium status and renal function requires further attention.

Plasma iron was also negatively correlated to the rapid decline in renal function in contrast to previous studies. For instance, a cross-sectional study conducted on the elderly population ≥ 90 years old in the areas of aged people in China found a negative correlation between plasma iron and CKD [15], which was consistent with our result. Nevertheless, another cross-sectional research on four rural regions in Hunan Province, China, observed no significant correlation between plasma iron and abnormal renal function [29]. A case-control study that recruited 79 age-matched volunteers in Sri Lanka showed that the iron levels in the CKD patient’s hair were greater than in controls [45]. Kidney iron metabolism is an extremely complex process, and iron deficiency and iron excess have some harmful health consequences. The median concentration of plasma iron (0.7 mg/L) in this study was lower than that in the general population (1.18~1.70 mg/L) [46,47,48], indicating the potential for iron deficiency in our population. This situation may explain the negative association between plasma iron levels and diminished renal function in our population.

### 4.3. Combined Impacts of Co-Exposure to Various Metals on the Decline in Kidney Function

The general population is co-exposed to various metals. However, the influences of that co-exposure cannot be assessed by the summed-up effects of single metals. There was a correlation in our study between the co-exposure to cadmium and lead to diminished kidney function measured based on fast kidney function decline or eGFR incident < 60 mL/min/1.73 m^2^. In single-metal analysis, we identified an association only between plasma cadmium and the decline in annual eGFR. Our findings indicated the potential for the unique impacts of co-exposure to various metals. Another longitudinal study in China also found a relationship between co-exposure to chromium with cadmium and lead to an additional decline in eGFR [49]. Cadmium and lead are the main soil contaminants accumulating in the body via the food chain. Cadmium has the ability to damage the redox balance in the cell and induce oxidative stress to affect organ systems [50], and lead has also been reported to inhibit calcium uptake by the mitochondria and impair oxidative metabolism in the kidney [51]. The shared mechanism of oxidative stress may result in the interactive toxic effects of cadmium and lead.

We identified the protective effect of iron and the harmful impact of chromium on renal function in single-metal analyses, thus finding the protective nature of the combined effects of co-exposure to these metals. Chromium induces toxicity in cells, DNA impairment, and oxidative stress in kidneys [52,53]. By contrast, iron supplementation could prevent nephrotoxic acute renal injury by inhibiting iron-catalyzed oxidative damage in iron-deficient mice [54]. Thus, the reduction in oxidative stress by adequate iron in the body may explain the negative connection between co-exposure to iron and chromium with the decline in renal function. A previous investigation also demonstrated that the antioxidant selenium may prevent or delay chromium-induced renal damage [55]. Namely, high levels of some protective metals may prevent the renal damage induced by toxic metals. However, the effect and mechanism of co-exposure to iron and chromium need further exploration.

### 4.4. Limitation

This research has some restrictions. First, only the total concentrations of metals were assessed without distinguishing valence states and metabolites. Despite using plasma components as the primary common biological markers, they may be inappropriate for assessing inner exposure to all components. Second, plasma metal concentrations were only assessed once at baseline. Nevertheless, because the individuals maintained their lifestyle, these values may still be accurate (in this situation, a single determination could serve as a snapshot of long-term exposure). Third, in comparison to the excluded individuals, the subjects enrolled in the final assessment were, to a great extent, male and had higher BMI, thus accounting for the observed high risk for the decline in kidney function in this study.

## 5. Conclusions

The kidney function decline was found to be positively correlated with plasma chromium and potassium, but negatively correlated with plasma selenium and iron in a midlife and aged community in China. In addition, the potential combined impacts of co-exposure to various metals were observed. Additional study is required to validate our outcomes and to illustrate the underlying mechanisms.

## Figures and Tables

**Table 1:** Initial characteristics of the study population (*n* = 1368).

Variables	Baseline	Fast Kidney Function Decline (Annual Decline in eGFR ≥ 5 mL/min/1.73 m^2^)			eGFR < 60 mL/min/1.73 m^2^		
		Yes	No	*p* Value	Yes	No	*p* Value
Subjects, *n* (%)	1368	408 (29.8)	960 (70.2)		189 (13.8)	1179 (86.2)	
Age (years)	57.6 ± 8.9	57.0 ± 8.8	57.8 ± 9.0	0.130	61.5 ± 8.4	56.9 ± 8.9	<0.001 *
Male sex, *n* (%)	754 (55.1)	212 (52.0)	542 (56.5)	0.126	82 (43.3)	672 (57.0)	<0.001 *
Marital status, *n* (%)				0.250			0.549
Married	1308 (95.6)	388 (96.5)	920 (95.8)		180 (95.2)	1128 (95.7)	
Single	54 (4.0)	14 (3.5)	40 (4.2)		9 (4.8)	51 (4.3)	
Smoking status, *n* (%)				0.424			0.125
Never smoker	815 (59.6)	249 (61.0)	566 (59.0)		125 (66.1)	690 (58.5)	
Current smoker	481 (35.2)	140 (34.3)	341 (35.5)		49 (25.9)	432 (36.6)	
Former smoker	72 (5.2)	19 (4.7)	53 (5.5)		15 (8.0)	57 (4.9)	
Drinking status, *n* (%)				0.772			0.003 *
Frequently	212 (15.5)	71 (17.4)	141 (14.7)		20 (10.6)	192 (16.3)	
Occasional	169 (12.4)	43 (10.5)	126 (13.1)		15 (7.9)	154 (13.1)	
Never	987 (72.1)	294 (72.1)	693 (72.2)		154 (81.5)	833 (70.6)	
Waist circumference (cm)	85.4 ± 8.6	85.6 ± 8.3	85.3 ± 8.8	0.593	85.8 ± 8.5	85.3 ± 8.7	0.469
BMI (kg/m^2^)	23.8 ± 3.1	23.8 ± 2.9	23.8 ± 3.2	0.960	23.8 ± 3.3	23.9 ± 3.1	0.586
Systolic blood pressure (mm Hg)	127.3 ± 16.1	125.8 ± 17.0	127.9 ± 15.7	0.026 *	127.0 ± 16.7	127.3 ± 16.0	0.803
Diastolic blood pressure (mm Hg)	79.0 ± 11.1	79.6 ± 10.7	78.7 ± 11.2	0.194	79.3 ± 10.6	78.9 ± 11.2	0.629
SCR (µmol/L)	71.9 ± 27.3	70.6 ± 12.3	71.7 ± 20.5	0.213	83.6 ± 33.1	69.4 ± 13.8	<0.001 *
FPG (mmol/L)	5.9 ± 1.4	5.9 ± 1.7	5.9 ± 1.3	0.551	5.8 ± 1.4	5.9 ± 1.4	0.448
TC (mmol/L)	4.8 ± 0.9	4.9 ± 1.0	4.7 ± 0.9	0.001 *	5.0 ± 1.0	4.8 ± 0.9	0.006 *
TG (mmol/L)	1.4 ± 1.1	1.4 ± 0.9	1.4 ± 1.1	0.340	1.5 ± 1.0	1.4 ± 1.1	0.350
HDL (mmol/L)	1.2 ± 0.2	1.1 ± 0.3	1.2 ± 0.2	<0.001 *	1.1 ± 0.3	1.2 ± 0.2	0.093
LDL (mmol/L)	3.1 ± 0.8	3.3 ± 0.8	3.0 ± 0.7	<0.001 *	3.3 ± 0.9	3.1 ± 0.7	0.001 *
Baseline eGFR (mL/min/1.73 m^2^)	91.6 ± 12.7	92.2 ± 10.5	91.4 ± 13.6	0.262	77.8 ± 13.5	93.8 ± 11.1	<0.001 *
Annual decline in eGFR (mL/min/1.73 m^2^)	2.9 ± 3.3	7.0 ± 1.5	1.2 ± 2.2	<0.001 *	6.3 ± 2.6	2.4 ± 3.1	<0.001 *

SCR: serum creatinine; FPG: fasting blood glucose; TC: total cholesterol; TG: triglycerides; HDL: high-density lipoproteins LDL: low-density lipoproteins; eGFR: estimated glomerular filtration rate. Continuous variables were represented as mean ± standard deviations (SD), while numbers and percentages were considered as categorical variables (%). *: Bonferroni-corrected statistical significance.

**Table 2:** Plasma metals’ concentration of different eGFR groups (*n* = 1368, median (interquartile range)).

Plasma Metals	Baseline	Fast Kidney Function Decline (Annual Decline in eGFR ≥ 5 mL/min/1.73 m^2^)			eGFR < 60 mL/min/1.73 m^2^		
		Yes	No	*p* Value	Yes	No	*p* Value
Lead (µg/L)	7.1 (3.0,15.4)	6.7 (2.0, 13.7)	7.2 (3.3, 15.9)	0.973	7.2 (3.1, 14.2)	7.1 (3.0, 15.4)	0.877
Cadmium (µg/L)	0.2 (0.1, 0.3)	0.2 (0.1, 0.4)	0.2 (0.1, 0.3)	0.090	0.2 (0.1, 0.4)	0.2 (0.1, 0.3)	0.068
Chromium (µg/L)	551.0 (391.5, 665.6)	580.9 (451.2, 686.0)	531.8 (366.8, 651.7)	<0.001 *	591.1 (459.3, 712.5)	541.2 (374.1, 660.5)	0.002 *
Copper (µg/L)	1129.0 (970.3, 1323.7)	1103.8 (935.4, 1280.0)	1138.7 (987.1, 1345.0)	0.001 *	1138.2 (965.3, 1323.9)	1127.1 (970.5, 1323.8)	0.804
Zinc (µg/L)	2389.0 (1880.6, 3207.1)	2485.8 (1907.5, 3348.8)	2332.7 (1866.6, 3133.3)	0.056	2568.3 (1909.5, 3449.1)	2360.1 (1870.3, 3174.0)	0.090
Selenium (µg/L)	22.0 (11.7, 117.34)	15.5 (10.0, 86.6)	53.1 (12.3, 125.3)	<0.001 *	17.7 (11.2, 97.9)	29.3 (11.7, 118.9)	0.028 *
Magnesium (mg/L)	20.2 (18.0, 22.8)	20.1 (17.8, 23.3)	20.2 (18.0, 22.7)	0.595	20.5 (18.1, 23.3)	20.1 (17.9, 22.7)	0.188
Iron (mg/L)	0.7 (0.3, 2.3)	0.4 (0.2, 1.5)	1.1 (0.3, 2.5)	<0.001 *	0.5 (0.3, 2.0)	0.8 (0.3, 2.4)	0.018 *
Calcium(mg/L)	98.8 (88.5, 111.2)	96.9 (85.7, 110.4)	99.5 (89.5, 111.4)	0.002 *	98.3 (85.6, 110.7)	98.8 (88.9, 111.2)	0.222
Sodium (g/L)	3.0 (2.7, 3.4)	3.1 (2.7, 3.6)	3.0 (2.7, 3.3)	0.001 *	3.1 (2.7, 3.6)	3.0 (2.7, 3.4)	0.195
Potassium (g/L)	0.8 (0.7, 1.0)	0.9 (0.8, 1.1)	0.8 (0.7, 1.0)	<0.001 *	0.9 (0.7, 1.1)	0.8 (0.7, 1.0)	0.001 *

eGFR: estimated glomerular filtration rate. *p* values were Bonferroni corrected. *: Bonferroni-corrected statistical significance.

**Table 3:** Difference (95%CI) in annual eGFR (mL/min/1.73 m ^2^) decline based on quartiles of primary components.

Component	Quartiles of Principal Components				*p*-Trend
	Q1	Q2	Q3	Q4	
PC1	0 (reference)	−0.20 (−0.70, 0.30)	−0.21 (−0.71, 0.29)	−0.11 (−0.61, 0.40)	0.436
PC2	0 (reference)	−0.38 (−0.87, 0.12)	−0.70 (−1.20, −0.20)	−1.54 (−2.03, −1.04)	<0.001 *
PC3	0 (reference)	0.99 (0.50, 1.48)	1.55 (1.06, 2.03)	2.02 (1.53, 2.51)	<0.001 *
PC4	0 (reference)	0.64 (0.15, 1.13)	1.08 (0.58, 1.57)	1.60 (1.10, 2.09)	<0.001 *

CI: confidence interval; PC: principal component. PC1: calcium, copper, and magnesium exposure pattern; PC2: iron and chromium exposure pattern; PC3: potassium and sodium exposure pattern; PC4: cadmium and lead exposure pattern. Adjustment of the models was set for age, gender, baseline eGFR, body mass index, smoking status, and drinking status. *: Bonferroni-corrected statistical significance.

**Table 4:** Adjusted ORs (95% CI) of swift renal function disorder and eGFR < 60 mL/min/1.73 m^2^ with an increase between the quartiles of each metal’s exposure pattern.

Component.	Q1		Q2		Q3		Q4		*p*-Trend
	OR (95% CI)	*p* Value	OR (95% CI)	*p* Value	OR (95% CI)	*p* Value	OR (95% CI)	*p* Value	
Fast Kidney Function Decline (Annual Decline in eGFR ≥ 5 mL/min/1.73 m^2^)									
PC1	1	1	0.76 (0.55, 1.06)	0.108	0.78 (0.56, 1.09)	0.150	0.84 (0.60, 1.17)	0.294	0.377
PC2	1	1	0.80 (0.58, 1.11)	0.183	0.64 (0.46, 0.89)	0.007 *	0.41 (0.29, 0.58)	<0.001 *	<0.001 *
PC3	1	1	1.59 (1.08, 2.32)	0.018 *	2.64 (1.84, 3.81)	<0.001 *	3.56 (2.48, 5.11)	<0.001 *	<0.001 *
PC4	1	1	1.14 (0.78, 1.65)	0.506	2.33 (1.64, 3.32)	<0.001 *	2.60 (1.82, 3.69)	<0.001 *	<0.001 *
eGFR < 60 mL/min/1.73 m^2^									
PC1	1	1	0.94 (0.57, 1.54)	0.802	0.80 (0.48, 1.33)	0.382	0.75 (0.45, 1.25)	0.272	0.975
PC2	1	1	0.60 (0.37, 0.98)	0.041 *	0.58 (0.35, 0.95)	0.029 *	0.42 (0.25, 0.71)	0.001 *	0.082
PC3	1	1	1.39 (0.79, 2.45)	0.255	2.23 (1.28, 3.88)	0.005 *	2.81 (1.64, 4.84)	<0.001 *	0.025 *
PC4	1	1	1.50 (0.87, 2.60)	0.146	1.72 (0.99, 2.98)	0.055	3.00 (1.75, 5.13)	<0.001 *	0.119

ORs: odds ratios; CI: confidence interval; PC: principal component; Q: quartile. PC1: calcium, copper and magnesium exposure pattern; PC2: iron and chromium exposure pattern; PC3: potassium and sodium exposure pattern; PC4: cadmium and lead exposure pattern; Adjustment of the models was set for age, gender, baseline eGFR, body mass index, smoking status, and drinking status. *: Bonferroni-corrected statistical significance.

## Data Availability

Not applicable.

## Supplementary Materials

The following supporting information can be downloaded at: https://www.mdpi.com/article/10.3390/toxics11030274/s1, Figure S1: Study flowchart.; Figure S2: Plasma metals’ factor loadings according to four principal components; Table S1: The results of quality asssurance (quality control); Table S2: Spearman correlation between plasma metals; Table S3: Demographic characteristics of included and excluded participants (age ≥ 45 years);
