# Supplementary material for: Combined Exposure to Multiple Metals and Kidney Function in a Midlife and Elderly Population in China: A Prospective Cohort Study
Source: Toxics. 2023 Mar 17;11(3):274. doi: 10.3390/toxics11030274 (PMC10051264; doi:10.3390/toxics11030274)
Supplement: Supplementary file 1 [file toxics-11-00274-s001.zip › toxics-2243075-supplementary.pdf]

Combined exposure to multiple metals and kidney function in a midlife and elderly population in China: a prospective cohort study

Tian-Ci Wang[1#], Li-Ming Zhang[2#], Yu-Jie Liu[1], Jian Li[2], Guo-Chong Chen[1], Hui Zhou[3], Lu-Gang Yu[3], Zhong-Xiao Wan[1], Chen Dong[1], Li-Qiang Qin[1*], Jing-Si Chen[1*]

[1]Department of Nutrition and Food Hygiene, School of Public Health, Suzhou Medical College of Soochow University, Suzhou, China.

[2]Suzhou Municipal Center for Disease Control and Prevention, Suzhou, China.

[3]Suzhou Industrial Park Centers for Disease Control and Prevention, Suzhou, China.

[#]Both authors contributed equally to this work

[*]**Corresponding to:**
Li-Qiang Qin, MD, PhD
Department of Nutrition and Food Hygiene, Suzhou Medical College of Soochow University, 199 Ren'ai Road, Suzhou 215123, China. Tel: 86-512-6588-0075; Fax: 86-512-6588-0050; Email: qinliqiang@suda.edu.cn

or
Jing-Si Chen, PhD
Department of Nutrition and Food Hygiene, Suzhou Medical College of Soochow University, 199 Ren'ai Road, Suzhou 215123, China. Email: jschen1993@suda.edu.cn

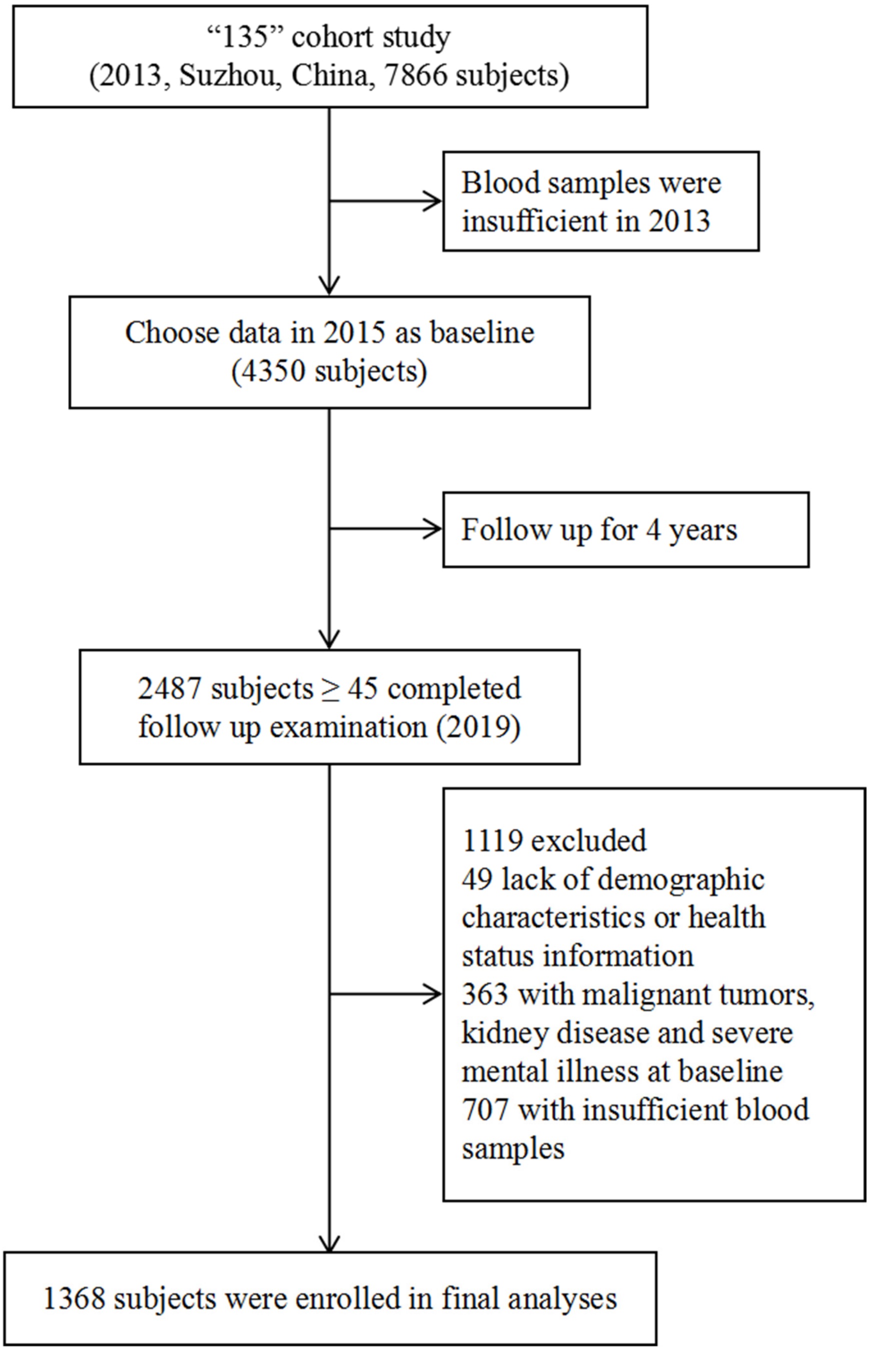

Supplementary Figure S1. Study flowchart.

Supplementary Table S1. The results of quality asssurance (quality control).

Elements	Isotope	LOD (μg/L)	Recovery (%)	RSD (%)	r
Lead	208	0.02	91.5	1.9	0.9993
Cadmium	111	0.01	97.6	1.8	0.9999
Chromium	52	0.40	101.0	1.3	0.9999
Copper	63	0.40	99.7	1.2	0.9999
Zinc	66	0.60	89.0	2.0	0.9998
Selenium	82	0.10	98.8	1.7	0.9992
Magnesium	24	0.30	102.5	1.4	0.9999
Iron	57	0.01	110.0	1.9	0.9998
Calcium	43	0.60	103.2	1.6	0.9994
Sodium	23	0.01	102.0	1.5	0.9996
Potassium	39	0.01	105.3	1.7	0.9998

LOD: limit of detection; RSD: relative sandard deviation.

Supplementary Table S2. Spearman correlation between plasma metals. (n=1368)

	Lead	Cadmium	Chromium	Copper	Zinc	Selenium	Magnesium	Iron	Calcium	Sodium	Potassium
Lead	1										
Cadmium	0.368**	1									
Chromium	-0.093**	-0.028	1								
Copper	0.147**	0.212**	0.310**	1							
Zinc	0.332**	0.291**	0.158**	0.251**	1						
Selenium	0.227**	0.164**	-0.201**	0.321**	0.086**	1					
Magnesium	0.122**	0.200**	0.368**	0.516**	0.296**	0.067*	1				
Iron	0.391**	0.329**	-0.044	0.327**	0.236**	0.708**	0.122**	1			
Calcium	0.282**	0.250**	0.361**	0.683**	0.438**	0.278**	0.717**	0.366	1		
Sodium	0.026	0.091**	0.200**	0.196**	0.183**	-0.170**	0.295**	-0.201**	0.335**	1	
Potassium	-0.072*	0.036	0.254**	0.020	0.129**	-0.226**	0.102**	-0.222**	0.072**	0.673**	1

*: $p<0.05$; **: $p<0.01$

Supplementary Table S3. Demographic characteristics of included and excluded participants (age ≥ 45 years).

**Characteristic**	**Included participants (n = 1368)**	**Excluded paricipants (n = 3621)**	***p* value**
**Age, n (%)**			
45-60	792 (57.9)	2308 (63.7)	0.177
> 60	576 (42.1)	1313 (36.9)	
**Sex, n (%)**			
Male	754 (55.1)	1564 (43.2)	< 0.001*
Female	614 (44.9)	2057 (56.8)	
**Marital status, n (%)**			
Married	1308 (95.6)	3457 (95.5)	0.558
Single	60 (4.4)	164 (4.5)	
**Smoking status, n (%)**			
Never smoker	815 (59.6)	2582 (71.3)	< 0.001*
Current smoker	481 (35.2)	938 (25.9)	
Former smoker	72 (5.2)	101 (2.8)	
**Drinking status, n (%)**			
Frequently	212 (15.5)	398 (11.0)	< 0.001*
Occasional	169 (12.4)	359 (9.9)	
Never	987 (72.1)	2864 (79.1)	
**BMI, median (IQR), kg/m^2**	23.7 (21.6, 25.9)	23.3 (20.9, 25.3)	0.038*

BMI, body mass index.

Plasma metals	Principal components			
	1	2	3	4
Calcium	0.920	-0.048	-0.196	-0.014
Copper	0.809	-0.020	-0.202	-0.234
Magnesium	0.799	-0.149	-0.062	-0.180
Iron	0.216	0.879	0.319	-0.014
Chromium	0.144	0.828	0.439	-0.084
Potassium	0.258	-0.345	0.775	0.188
Sodium	0.452	-0.392	0.637	0.150
Cadmium	0.089	0.095	-0.159	0.727
Lead	0.261	0.179	-0.311	0.640
Zinc	0.354	0.057	0.063	0.134
Selenium	0.340	0.182	-0.450	-0.100

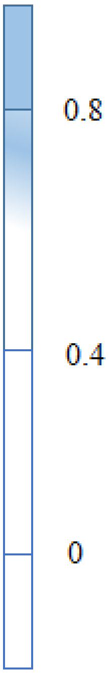

Supplementary Figure S2. Plasma metals' factor loadings according to four principal components (n=1368).
